# Phenotyping Women Based on Dietary Macronutrients, Physical Activity, and Body Weight Using Machine Learning Tools

**DOI:** 10.3390/nu11071681

**Published:** 2019-07-22

**Authors:** Ramyaa Ramyaa, Omid Hosseini, Giri P. Krishnan, Sridevi Krishnan

**Affiliations:** 1 Department of Computer Science, NMT Computer Science and Engineering, Socorro, NM 87801, USA; 2 Department of Medicine, University of California San Diego, San Diego, CA 92093, USA; 3 Department of Nutrition, University of California Davis, Davis, CA 95616, USA

**Keywords:** phenotyping, machine learning, energy balance, macronutrients, postmenopausal women

## Abstract

Nutritional phenotyping can help achieve personalized nutrition, and machine learning tools may offer novel means to achieve phenotyping. The primary aim of this study was to use energy balance components, namely input (dietary energy intake and macronutrient composition) and output (physical activity) to predict energy stores (body weight) as a way to evaluate their ability to identify potential phenotypes based on these parameters. From the Women’s Health Initiative Observational Study (WHI OS), carbohydrates, proteins, fats, fibers, sugars, and physical activity variables, namely energy expended from mild, moderate, and vigorous intensity activity, were used to predict current body weight (both as body weight in kilograms and as a body mass index (BMI) category). Several machine learning tools were used for this prediction. Finally, cluster analysis was used to identify putative phenotypes. For the numerical predictions, the support vector machine (SVM), neural network, and k-nearest neighbor (kNN) algorithms performed modestly, with mean approximate errors (MAEs) of 6.70 kg, 6.98 kg, and 6.90 kg, respectively. For categorical prediction, SVM performed the best (54.5% accuracy), followed closely by the bagged tree ensemble and kNN algorithms. K-means cluster analysis improved prediction using numerical data, identified 10 clusters suggestive of phenotypes, with a minimum MAE of ~1.1 kg. A classifier was used to phenotype subjects into the identified clusters, with MAEs <5 kg for 15% of the test set (n = ~2000). This study highlights the challenges, limitations, and successes in using machine learning tools on self-reported data to identify determinants of energy balance.

## 1. Introduction

Postmenopausal women readily gain body fat and weight, along with facing challenges in reducing or maintaining a healthy body weight. [1]. This puts them at a higher risk for chronic cardiometabolic diseases. More so than premenopausal women, overweight or obese postmenopausal women are at an increased risk for cancer, cardiovascular disease, and type 2 diabetes [2]. Studies on postmenopausal women indicate that body weight can be maintained by reducing energy intake, however, this is not successful for all women [3]. Several factors determine body weight in postmenopausal women, and these factors are highly variable across the population [4]. This inter-individual variability needs to be understood and quantified in order to be able to help weight loss or maintenance programs in this at-risk population. One way to understand this inter-individual variability is to phenotype body weight regulation using determinant independent variables. The classic “phenotype” is “the set of observable characteristics of an individual resulting from the interaction of its genotype with the environment” [5]. Using this approach, a nutritional phenotype could be “a set of observable characteristics of an individual resulting from the interaction of their ‘diet’ with their ‘environment’”. In the present work, we use this definition of phenotyping to focus on understanding the relationship between (a) macronutrient and energy intake, (b) physical activity, (c) sociodemographics, (d) other disease states, and (e) body weight. 

Within the framework of phenotyping, data analysis plays a significant role [6]. Statistical prediction models have been used in nutrition as early as the 1900s. The earliest and simplest examples are based on classical linear regression analyses, such as the Harris–Benedict equation [7], or the more recent Mifflin St.Jeor equation [8]. These are well known tools used in energy metabolism. These regression models ascribe to the one-size-fits-all theme that has been largely used in nutrition science. In recent years, as the dimensionality of data has increased, more complex tools have become necessary to build predictive models. Personalizing efforts using more complex means of statistical analysis are becoming more common [9]. The approach has also changed from focusing on a “mean” outcome to “individualized” outcome predictions, adding to the complexity. 

Our objective was to be able to identify subpopulations or ‘phenotypes’ within a large population of postmenopausal women from the Women’s Health Initiative-Observational Study (WHI OS), based on the relationship between body weight and dietary macronutrients, physical activity, and socioeconomic variables. Machine learning has been used in the past to determine dietary factors that are associated with a risk of weight gain in postmenopausal women from WHI OS [10], however, we took a different approach. We trained machine learning algorithms on the macronutrient composition of diets and physical activity, as well as other pertinent demographic data, to predict current body weight. Our goals were as follows: (a) To understand which commonly used algorithms achieve the best prediction and why they do so; (b) to understand how these algorithms group women into clusters that might reflect underlying phenotypes, and finally; (c) to evaluate how the relationship between dietary, physical activity and sociodemographic variables to body weight is different between these clusters. This paper presents several such models, and their evaluation to identify ideal prediction algorithms for the given data, based on their ability to predict body weight, both numerically, as well as categorically (i.e., as body mass index categories). In addition to these objectives, this is a crucial exercise in understanding the inherent limitations to self-reported data and how they influence model building using novel and recently introduced transdisciplinary approaches. Further, efforts to accurately identify macronutrients able to predict body weight in postmenopausal women could reduce the disease burden in this population. 

## 2. Methods

### 2.1. Data Acquisition

We obtained data from the Biological Specimen and Data Repository Information Coordinating Center (BioLINCC), an online repository of epidemiological and clinical data hosted by the National Library of Medicine. We used the data from the Women’s Health Initiative Observational Study (WHI OS). This is a long-term national health study on postmenopausal women which was started in 1991. The primary aim of this study was to identify strategies to prevent breast and colorectal cancer, heart disease, and osteoporosis in postmenopausal women. There were two components that were initially started as part of the Women’s Health Initiative (WHI): The randomized controlled clinical trial (CT) and the observational study (OS). The WHI OS was aimed at observing and analyzing how well lifestyle behaviors such as diet, exercise, and prior disease risk factors predicted disease outcomes, primarily heart disease and cancer. Enrolment for the WHI OS started in 1994, and the study was completed in 1998. A total of 93,676 women were recruited for this study. The selection criteria for recruitment was women that were postmenopausal, between 50–79 years of age, with the ability and willingness to provide the information at baseline and follow up visits, and those who were planning to reside in that area for a minimum of 3 years. For the sake of answering our primary question we used the WHI OS data. Data that were of interest to our question were available from the baseline study. As part of the WHI OS, women answered a modified semi-quantitative Block food frequency questionnaire in 1994 about their dietary intake between the years of 1993 and 1994. In addition, they also filled out a physical activity questionnaire that categorized their activity levels into mild, moderate, and vigorous physical activity [11]. Further, we obtained information about their health status, such as the presence of diabetes or hypertension, and demographic information, such as their age, ethnicity, education, income, and marital status. Education and income were combined to arrive at one socioeconomic score based on the method devised by Green L.W. in 1970 [12]. The Institutional Review Board (IRB) at UC Davis and New Mexico Technological Institute exempted the review and approved use of the data. All data treatment and analyses were done in Microsoft Office Excel (Redmond, WA, USA) JMP Pro 14.1 (Cary, NC, USA), R version 3.1.1 [13], Python [14], and Jupyter notebooks [15].

### 2.2. Data Cleanup

**Figure 1:**
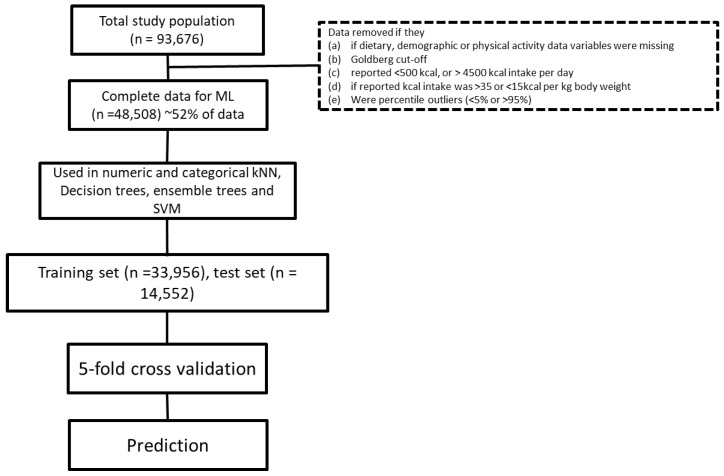
Flow chart presenting the data cleaning and preparation process, including the machine learning (ML) algorithms applied. kNN—k-nearest neighbors, SVM—support vector machine.

The Goldberg cutoff, which reduces the likelihood of implausible energy intake reports, was calculated as described earlier [16,17]. This cutoff scales the self-reported energy intake to the estimated basal metabolic rate (Mifflin St. Jeor equation [8]), matched to their physical activity level, estimated from their self-reported physical activity. Beyond this, results where the total energy intake was reported to be <500 kcal/day or >3500 kcal/day were considered outliers and removed from the dataset. We only considered data where the self-reported energy intake per kg of body weight was between 15–35 kcals. In addition, we also used a percentile method to remove outliers. We removed data if they were less than the 5th or greater than the 95th percentile for that data range [18]. These outliers were removed from the dataset to ensure the development of a robust model. In the final analysis, data from 48,508 subjects were used to train the algorithm (“known data”) to predict the weight of 14,552 “test” subjects. Figure 1 depicts the process of final study volunteer identification. 

### 2.3. Feature Selection

The dietary data collection methods, their implementation, and the obtained data variables from the WHI OS study are discussed here [11]. An extensive feature selection process was used, which included standard least square regression, stepwise multiple linear regression, partial least squares (PLS) regression and variable cluster analyses, returning a total of 29 variables with a *p* < 0.0001 significance of being associated with body weight. For this first pass approach, 140 dietary variables, 12 anthropometric variables, and 3 physical activity variables, as well as chronic disease state information were used. However, since our objective was to use macronutrients pertinent to body weight, from amongst these selected features, only the variables of physiological relevance were chosen to represent their diet, such as total energy (kcals), dietary fat (g), dietary protein (g), dietary carbohydrates (g), dietary sugars (g), dietary fiber (g), and alcohol (g). In addition, mild, moderate, and vigorous intensity physical activity, height, socioeconomic score, and marital status were retained in the final model. All disease state information was also retained in the final model, to adjust for the given disease. 

### 2.4. Data Preprocessing

Normalization: The input features were in various units, with the mean values spanning several orders of magnitude. Many machine learning (ML) algorithms are very sensitive to differences in the scale of magnitudes of different inputs. To address this, we normalized the data, using a z-score transformation ((value−mean)/standard deviation).

Principal Component Analysis: Principal component analysis (PCA) converts a set of observations of correlated variables into a set of values of linearly uncorrelated variables called principal components. Using an orthogonal transformation does this. The number of principal components is less than or equal to the smaller of the number of original attributes. This transformation is done in a way that the first principal component has the largest possible variance, and each succeeding component, in turn, has the highest variance possible under the constraint that it is orthogonal to the preceding components. We did PCA on our input features as a way of dimensionality reduction, and to increase independence among the inputs to the machine learning algorithms. We performed PCA on the independent variables, and used the first 15, which explained 97.9% of the variance, in the feature set. This was done so that (a) the number of features were reduced (thus reducing the ‘curse of dimensionality’ which affects most of the ML algorithms), (b) mutually correlated features were not used as inputs, and (c) so that the residual variance in the data (which might be noise) would not be modeled. 

In order to be able to report on how classical regression tools such as stepwise multiple linear regression perform in comparison to machine learning-based tools, we compared them side-by-side. Stepwise multiple linear regression was done using Akaike information criterion (AIC) as the decision criteria (a lower AIC represents a better fit), in a mixed approach (the combination of backward and forward, deletion and addition (respectively) of independent variables to the model to determine the best fit). 

### 2.5. Numerical Prediction

Predictive modeling was carried out using machine learning algorithms: For numerical prediction, weight in kg was predicted from the input variables. Simpler ones such as statistical regression and regression tree did not yield good results (data not shown). Neural networks and support vector machines (SVMs, explained below) are powerful techniques which are capable of learning complicated functions. The method that worked best for these data was a local, instance-based learning method called k-nearest neighbors (kNN, explained below).

### 2.6. Regression SVM

Support vector machines or support vector networks are supervised learning algorithms that are commonly used when input vectors are non-linearly mapped to a very high dimensional feature space, that has a linear relationship to the output. This ensures high generalizability of the learning machine [19]. The algorithm uses a kernel function to measure similarities between two different patterns by returning a real value. One simple, but not sufficiently general way to measure similarity in a kernel function is by using the canonical dot product. Vapnik (1995) presented an ε–insensitive loss function to compute SVM regression. 

This method is very powerful for non-linear analysis, but the search space in which to find the non-linear mapping becomes too large for high-dimensional input data, posing a practical challenge in use [20,21].

### 2.7. Neural Network

Neural networks are biologically inspired algorithms, consisting of a large number of very simple and independent processing units (neurons) which are connected unidirectionally. Neural networks are a powerful tool to capture the semantics or dynamics of factors that linked by highly nonlinear functions. 

The first neural network model was designed by McCulloch and Pitts in 1946 [22]. Rosenblatt introduced the first perceptron model and discussed its convergence to correct weights [23,24,25]. Parkers and Rumelhart et al. introduced the back-propagation multilayer neural network model for weight determination [26,27]. The back-propagation algorithm for nonlinear least squares uses feed-forward neural networks for training. Compared with a conjugate gradient and variable learning rate algorithms, the back-propagation algorithm is much more efficient when the network contains no more than a few hundred weights, and also in many cases, the algorithm converges while the aforementioned algorithms fail to converge [28,29]. For this reason, in the present study, this algorithm was chosen. 

### 2.8. k-Nearest Neighbors

Instance-based learning approaches, such as the k-nearest neighbors (kNN) algorithm, follow a straightforward way to approximate real or discrete valued target functions [30,31]. The learning process is lazy and consists of storing training data. Predicting the output of a new input vector involves fetching similar instances from the saved training data and aggregating their outputs. Unlike many other techniques that build only one local approximation to the target function, one significant advantage of instance-based algorithms is that for each new query instance the model can build a new approximation to the target function. This gives instance-based algorithms, specifically case-based algorithms, the ability to capture very complicated relationships between attributes and outcomes. There are two big disadvantages associated with the instance-based approach: (i) The cost of classification is much higher than with other methods, since all computations are performed at the classification time rather than while training; and (ii) this method incorporates all attributes of the instances when the algorithm tries to bring back similar training examples from memory. If the target variable is only dependent on a few of the attributes, this can cause instances that are very similar to be predicted further apart with a large distance [32,33]. 

In the k-nearest neighbors method, which is the most basic algorithm among instance-based methods, all instances are mapped to points in n-dimensional space ($R^{n}$). Different distance measurement techniques can be applied to calculate nearest neighbors. The original algorithm uses the standard Euclidean distance method. It should be noted that Euclidean distance and square Euclidean distance are usually used when data are not normalized. These two methods are also very sensitive to the scale of different independent attributes, and having one or more attributes with a large scale can decrease the effect of other attributes [34]. The city block (Manhattan) distance between two independent attributes, unlike the Euclidean distance, is measured as the distance along the *x*-axis plus the *y*-axis. For the numeric kNN method, 75% of the dataset was assigned to the training subset and the remaining 25% was assigned to testing. To validate the models, 5-fold cross-validation was used. 

### 2.9. Information Gain and Loss Models

In order to study the effect of each independent variable on the outcome accuracy, all independent variables were excluded from the model one by one and a new model was developed based on the other independent variables. Three different sets of models were observed, based on the effect of variable exclusion: (a) Removing variables that improved the accuracy, (b) removing variables that had no specific effect on outcome accuracy, and (c) removing all variables in (*a*) and (*b*). 

### 2.10. Categorical Prediction

Here, independent attributes were used to predict body mass index (BMI) categories (underweight (BMI<18.5), normal weight (18.5≤BMI<25), overweight (25≤BMI<30), grade I obese (30≤BMI<35), grade II obese (35≤BMI<40), and grade III obese (40≤BMI)). For a categorical approach, bagged trees, decision trees, SVMs, kNNs, and ensemble trees were evaluated (as mentioned before, we do not present methods that were not promising).

### 2.11. Decision Tree

Decision tree learning algorithms are one of the most effective and widely used inductive inference methods for discrete valued target functions [30]. A decision tree is a tree structure of Boolean questions about the input variables, with each branch ending in a leaf marked by an output category. If the input variables are such that a particular branch would be traversed, the corresponding leaf is the predicted classification of BMI. 

A decision tree learning algorithm is a top-down, greedy algorithm, which constructs the tree as follows: Question the input attribute that has the most mutual information with the output, then ask the question that will reduce the information content (entropy) of the output. This process is repeated for each branch corresponding to each answer of the question. The ID3 decision tree algorithm was used for this dataset. 

### 2.12. Ensemble Methods

In order to improve the generalizability and robustness of a predictor, the results obtained from several basic predictors of a given learning algorithm can be combined. Ensemble methods try to combine the obtained results to achieve this goal. Ensemble methods can be categorized into two general groups: (a) Averaging methods, such as bagging methods or forests of randomized trees, or (b) boosting methods such as AdaBoost or gradient tree boosting [35].

Bootstrap aggregating (bagging) is a machine learning ensemble meta-algorithm designed to improve the stability and accuracy of algorithms such as decision trees, neural networks, and linear regression models. Generally, bagging predictors is a method of using multiple versions of base predictors to obtain an aggregated predictor. When the outcome is discrete-valued, the aggregation uses averaging over all different versions, while for a categorical outcome it uses a plurality vote to obtain the best accuracy. One of the main problems related to prediction methods is instability. Bagged methods could increase stability when altering the learning set, making a huge difference in the constructed predictor [36]. The major difference between bagging methods arises from how the random subsets of training data are chosen. [35].

Boosting methods follow a different logic process than bootstrap aggregating, where many weak models are combined to produce an ensemble model that is more powerful. The base predictors are generated sequentially and one tries to reduce the bias of the combined predictor [37]. One of the most popular boosting algorithms is AdaBoost, which was introduced by Freund and Schapire in 1995 [38]. The algorithm uses many weak learners which perform/predict slightly better than random guessing. Following this, it fits these weak learners repeatedly on the data and then uses the weighted majority vote or sum technique to combine the results for the final prediction.

### 2.13. Validation

For all machine learning methods, there is always the risk of overfitting or underfitting. Overfitting happens when the model is too complex for the data, and this is due to the small size of the dataset or the presence of too much noise in the data. In the case of overfitting, the complex generated model captures the noise in training dataset. When overfitting happens, the algorithms show a low bias (error) and high variance. In contrast, underfitting occurs when the statistical model or machine learning algorithm cannot capture the underlying trend of the data and consequently the model does not fit the data correctly. In the occurrence of underfitting, high bias and low variance are obvious.

Overfitting has problematic consequences for machine learning algorithms and leads to poor predictions for unseen input instances. Validation methods are essential tools in a machine learning algorithm that are used to make sure the constructed model is suffering from neither underfitting nor overfitting. A very basic approach, called the validation set method, is randomly dividing the dataset into training and testing (70% training and 30% testing) subsets, then building the model based on the training data, and then testing the model’s performance with the testing subset. While this method is very fast and easy to implement, it also has some drawbacks. The testing error can be highly variable depending on which observations are included in the testing and training datasets. The other problem related to the validation set method is that the model is developed on only a subset of the data, and this potentially can lead to a higher estimation error and consequently a poorer model in the test phase. 

To address the problems related to the validation set method, cross-validation tries to create a test dataset during the training phase by partitioning the dataset into subsets and using one subset, called the validation dataset, for testing and the rest for training purposes. There are different cross-validation methods, such as leave one out (L-O-O) and k-fold validation. In the L-O-O method, the model is being trained on n−1 observations and is validated on the single validation set observation. The test error based on only one single observation is highly variable, however, if the process is repeated for all instances in the dataset, then the average of all these test errors gives the overall error of the dataset. Notably, having less bias in the regression coefficients and no variation in parameter estimations across the training dataset are two big advantages of this method, however, for large datasets it is computationally expensive. 

The k-fold cross validation method is a compromise between the two aforementioned methods. This was the method used in the present work. The dataset is randomly divided into k subsets (also known as a fold). One subset is used to test the model and the other k−1 subset is used to train the model. This process is repeated k times for all subsets and the average of the these k test errors presents the overall error of the dataset. This method requires less computational resources and the estimation is more accurate than what is produced with the L-O-O method (the L-O-O method has less bias than k-fold cross validation but possesses a larger variance) [20]. 

### 2.14. Clustering

Powerful ML methods such as neural networks (NNs) and SVM can fail for many reasons, including the presence of many dimensions, potentially irrelevant ones, too much noise, etc. For our dataset, the training set error was quite high, indicating that the desired function was not being learned. This fact, coupled with the fact that local and ensemble models perform well, led to our conjecture that for similar values of inputs we had widely varying outputs. In such cases there may be hidden variables which determine the outputs, or the function from input to output. If this were the case, clustering would improve the performance of the algorithms. 

Cluster analysis groups sets of objects that have maximal similarities. Hierarchical clustering analysis (HCA) or connectivity-based clustering follows the simple idea that the nearby objects are more related to each other than the ones further away. HCA, which is a greedy algorithm, falls into two different categories: Agglomerative (linkage) and divisive (K-means and K-medoids). In agglomerative (bottom-up) clustering, each object starts in its own cluster and a pair of objects can merge as one moves up, however, in divisive cluster analysis, which is a top-down method, all objects are in one cluster and different clusters disjoin recursively as one moves down.

K-means is the simplest and most commonly used algorithm which uses a squared error criterion. This algorithm represents clusters by their centroids, which is the mean of all objects in one specific cluster. The algorithm for K-means clustering uses the partitioning error to stop the iteration. This algorithm can be presented as a gradient-descent procedure, which starts with an initial fixed number of cluster centroids, constantly updating them to reduce the error as much as possible. The K-means algorithm has a linear time complexity, and this makes the algorithm popular for researching large datasets [39]. 

The performance measures of clustering algorithms quantify the proximity of points in each cluster and the distance separating points from different clusters. There are several measures, and they all measure the distance between each data point from the centroid of its cluster (as a measure of cluster cohesiveness) and the distance between centroids of various clusters (as a measure of cluster separation), combining the two measures. 

The clustering algorithms we used were sensitive to the number of clusters, which is a parameter specified by the user during the initial random clustering. The clustering algorithms are very fast, and as such, they can be run many times with many different initializations for each desired cluster number, where a maximum value of the desired criteria of inter-cluster separation and intra-cluster cohesion is produced. We used this process, repeatedly varying the number of clusters to determine the ideal number of clusters. The Calinski-Harabasz criteria was used to measure the effectiveness of clustering [40]. We used this measure, along with the average prediction error, to determine the number of clusters. A higher Calinski-Harabasz score represents a lower intra-cluster distance and an increased inter-cluster distance.

### 2.15. Phenotyping by Predicting Subjects into Identified Clusters Using a Classifier

Clustering generally improves the performance of machine learning methods, however, for an unseen, newly added case/data point/subject, it is challenging to predict which cluster it could belong to. In order to address this, we developed a three-step model where we first tried to predict the cluster that a new data point should belong to, then use that cluster to predict their body weight. This effort would both validate the clusters identified, as well as determine the predictive ability of the model to consistently classify a specific set of independent variables to the same cluster, thereby establishing a “phenotyping” effort. Finally, we added a third step to improve the number of points predicted, as described below.

Step 1: For each cluster, we developed a machine learning model (neural network with 1 hidden layer and 15 neurons) that would evaluate whether the new case’s independent variables belong to the given cluster. The neural network was trained with a training set which inputs the independent variables, deciding between a result of “in the cluster” or “not in the cluster”, based on whether the cluster could predict the case’s weight within a predetermined threshold (by varying this threshold we can control how strict the network’s admittance of a case into a cluster is). Therefore, a new case’s independent variable list is fed into the neural networks of all the clusters and they all predict whether the data point might belong to their cluster. Since this is a new data point there is error associated with this prediction. This error translates into multiple clusters agreeing to “admit” the new case or none of the clusters agreeing to “admit” it. 

Step 2: Each cluster that included the new case predicts the body weight of the case based on the independent variables, and an average of these predictions is computed, along with an error term for the predicted weight. The prediction within the cluster was done via SVM or kNN. If no cluster “admits” the case, the body weight for that case cannot be predicted via clustering. As expected, we saw that when the ML algorithms for admittance into clusters were trained to be conservative, the overall error associated with the predicted body weight was low, but the number of cases predicted was also low. 

Step 3: If a case was not included in any of the clusters, the prediction from the larger kNN model was used. 

## 3. Results 

The independent variables that were used to predict the body weight and BMI category are listed in Table 1.

### 3.1. Numeric Prediction Models

**Figure 2:**
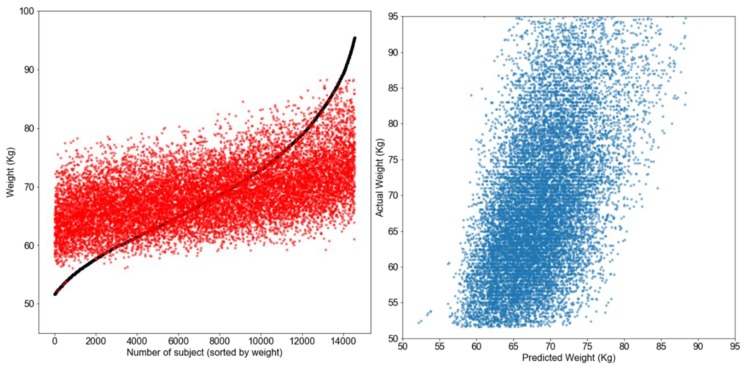
The **left panel** shows a scatter plot comparing the predicted and real weights against the weight index. The **right panel** shows a comparison between the predicted weights by kNN against the real weights. In the left panel, the red dots are the predicted weights and the black squares are the real weights.

Table 2 summarizes the results from different models evaluated for numerical prediction using the independent variables. Gaussian SVM regression performed the best, with a mean approximate error (MAE) of 6.70 kg and a $R^{2}\;$of 0.30. The two-layer feed forward neural network trained model was a close second (MAE = 6.90 kg and $R^{2}$ = 0.27), and kNN (18 neighbors) performed the next best (MAE = 6.98 kg and $R^{2\;}$ = 0.26). Figure 2 shows a scatter plot that presents the relationship between the predicted weights versus the real weights, as well as the distribution of errors in prediction for the kNN model.

### 3.2. Categorical Prediction Models

**Figure 3:**
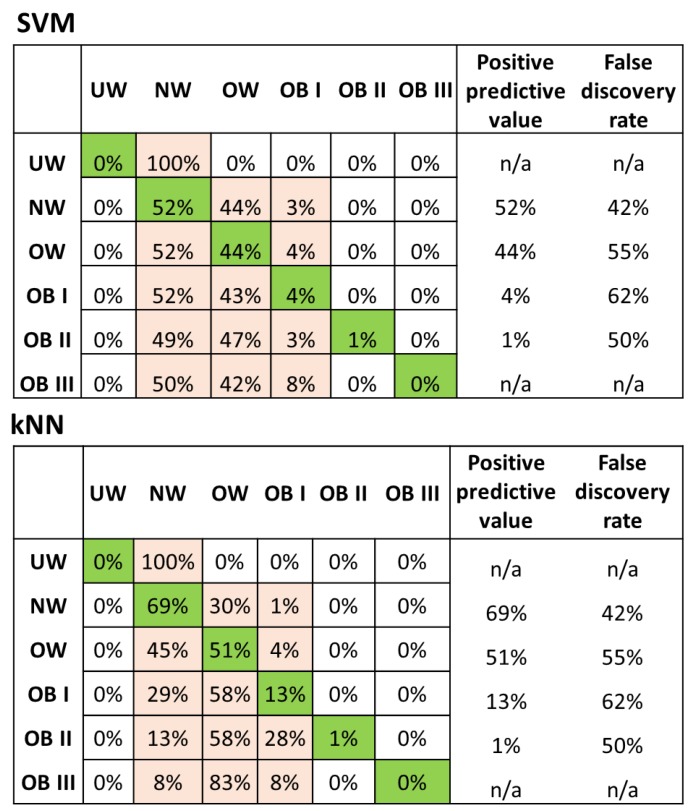
Confusion matrix for the ensemble tree with the subspace method, using nearest neighbor learners. Green color boxes indicate positive predictions and cream color boxes indicate false predictions.

Similar to the numerical prediction of weight, the categorical SVM algorithm had the best accuracy for BMI classification (54.5% accuracy, note that a random prediction among six categories will have an accuracy of 1/6 or 16.7%). Ensemble trees, using nearest neighbors as the learner type, were also able to predict the BMI category with a 52.8% accuracy, closely followed by actual kNN (51.9% accuracy), while decision tree models had the worst fit (44.2% accuracy). This is presented in Table 2, along with the implementation details for the model. Figure 3 displays the confusion matrix, outlining the positive predictive value, as well as the false discovery rate for the categorical kNN and SVM regression models (the random forest and bagged tree results were very similar, data not shown). The normal weight BMI category had the highest positive predictive value (69%), while the underweight, grade II, and grade III obese categories had the lowest (0–1%) positive predictive values. The highest misclassifications were also in these categories: 58% of grade I and II obese were misclassified as overweight, and 83% of grade III obese were classified as overweight as well. 

### 3.3. Information Loss and Gain Model Evaluations

We evaluated the effect of elimination of each independent variable on the MAE, $R^{2}$ and RMSE values (Table 3). Removing different variables did not have a significant impact on the MAE, $R^{2}$, and RMSE values, and empirically speaking, these may be slightly worsened by the model fit. Dietary fiber had the least impact on being removed from the model (MAE = 6.99 kg and $R^{2}$ = 0.27), while dietary protein had the highest impact and worsened the model fit (MAE = 7.09 kg and $R^{2}$ = 0.25), albeit these are all very modest changes. Removing dietary fat or carbohydrates did not impact the model significantly. 

### 3.4. Cluster Analysis

**Figure 4:**
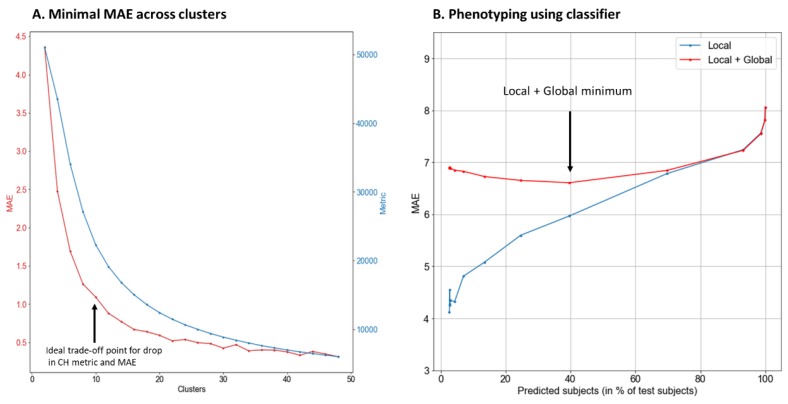
(**A**) Plotting the MAE (left y-axis, red line) and Calinski-Harabasz score (right y-axis, blue line) against number of clusters (x-axis). After 10 clusters, the reduction in MAE and the score tapers off, suggesting that could be a good number of clusters for further evaluation. The difference in MAE and Calinski-Harabasz score between 10 clusters and 40 clusters is <0.5 kg and ~10,000 points, which is much less compared to 2–10 clusters (from 4.3 kg and 50,000 points). (**B**) Plotting the change in MAE (y axis) and the percentage of predicted test subjects (x-axis). The red line represents the MAE for the combined global and local models and the blue line represents the MAE of only the local model. The different percentages cases that were predicted were obtained for different conditions (local and local and global together) by changing the criteria for the training set of the classifier that predicts the cluster of each case.

While global models (SVM and neural networks) performed modestly when predicting body weight, local models, either by themselves (kNN) or within an ensemble (kNN as a learner for random forest), performed just as well. However, neither were able to predict the body weight as closely as could be expected. So, cluster analyses were carried out to see if they could improve the model fit and prediction. A local unsupervised learning clustering tool (K-means clustering) was used to identify ‘phenotypes’ within this population. The K-means cluster used the Calinski-Harabasz score, as mentioned earlier. We found that an increase in the number of clusters decreased both the Calinski-Harabasz score and the error. So, we chose to increase the number of clusters, so long as the drop in error matched the drop in the Calinski-Harabasz score. After 10 clusters, the drop in the Calinski-Harabasz score was not compensated by the drop in error, so we consequently chose to use 10 clusters (Figure 4A). 

Once the clusters were identified, kNN was used again to predict body weight within each cluster (kNN and SVM performed similarly, and the risk of overfitting is considerably less with kNN, owing to its simplicity, so the data presented are for kNN). A summary of fit characteristics associated with these clusters is given in Table 4. It is important to note that once the clusters were formed, kNN performed significantly better within each cluster at predicting body weight using the same input variables (MAE = 1.1 kg), rather than using the population as a whole (MAE = 6.98 kg). The variance within each cluster is smaller, which makes the r values smaller, but the prediction more accurate. 

### 3.5. Phenotyping Using Classifier

The results from the classifier, used to identify which cluster each new case fitted into from the test set, are presented in Figure 4B. This classifier was trained as follows: For each cluster, a separate one-class classifier was developed (i.e., this will say ‘yes’ for only one-class and ‘no’ to all others); The training data was a subset of the entire data used in clustering. Training set for one-class classifier for cluster k only contained those data points whose error was <3.5kg when the KNN of cluster k was used to predict its weight. (We varied the error value as a parameter, which is shown in Figure 4B. For approximately 2000 participants, the cluster classifier was able to accurately place them within their body weight cluster with a MAE of <5 kg. The higher the permissible error in prediction, the more successful was the prediction. If it was lower, the success rate was lower, but the error associated with the prediction was lower. 

**Figure 5:**
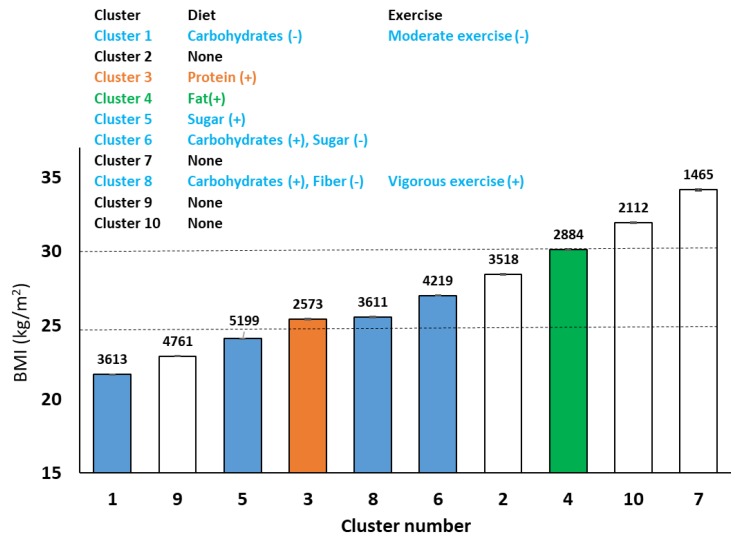
Clusters and their BMI classes, along with the number in each cluster. The inset table shows that stepwise multiple regression analysis identified the strongest predictors of body weight (kg) based on macronutrient and exercise subcomponents. Positive and negative signs indicate the directionality of association.

Step-wise multiple regression (AIC, mixed) was used to determine which macronutrient and physical activity variables had a significant relationship with body weight, as well as their directionality within each cluster. The variables/parameters listed were significantly associated (*p* < 0.05) with the outcome variable (body weight), after false discovery rate (FDR) correction. These results are summarized in Figure 5. Clusters 2, 7, and 9 were not identified to have predictors that were macronutrients or physical activity. Cluster 3 indicated a strong positive association between body weight and protein. Clusters 1, 6, and 8 displayed strong associations between dietary carbohydrates and body weight, and while there was an inverse association in cluster 1 (the lowest body weight), there was a positive association for clusters 6 and 8. Cluster 5 showed a strong positive association between dietary sugar and body weight, but not total carbohydrates. Dietary fiber was inversely associated with body weight in cluster 8, and vigorous intensity physical activity was positively associated. Cluster 4 displayed a positive association between body weight and dietary fat. 

## 4. Discussion

To our knowledge, this is the first effort to predict current body weight using machine learning tools from self-reported dietary macronutrient and physical activity data, adjusting for sociodemographics and disease states. Our effort was focused on using modeling algorithms as both predictive and inferential tools. This study identified that SVM regression was the best suited tool for this task, closely followed by neural network and the k-nearest neighbor algorithms. While the overall data model showed a reasonable fit and predictive ability, clustering produced relatively superior fit statistics.

The primary rationale for predicting current body weight based on current macronutrient intake is a crucial first step for being able to accurately predict body weight change. Without understanding the dynamics of how self-reported macronutrient intake predicts present body weight, going on to understand how a change in dietary intake would affect body weight becomes unwieldy. Furthermore, this is the first time the feasibility of using self-reported data to accurately predict body weight was evaluated, with modest success. 

SVMs and neural networks are very powerful methods and are capable of learning in most cases [41,42]. The modest fit seen in this data points to the fact that there is not a global function that can be learned easily, likely owing to a combination of variability, as well as noise (i.e., errors in reporting). Further, in cases where powerful methods such as SVMs and neural networks perform comparable to simple ones (such as kNN), data inherently satisfies the assumptions of the simpler method. This is comparable to an instance where cubic regression performs just as effectively as linear regression, suggesting the relationship is likely linear. The simple method that works well is kNN, which groups data points into neighborhoods, suggesting that the data contains many categories of ‘associations’ between independent variables and weight. The method is a local model that forms a simple interpolative model around the neighborhood of each data point. It is a type of non-parametric modeling, where a single predefined model from a predefined template is not expected to fit the entire dataset. Parametric model fitting algorithms assume a predefined model template and model fitting involves determining the parameters of the model template that best fits the data [43]. For instance, linear regression assumes the model to have the template of a linear equation, attempting to find the coefficients of the equation. Non-parametric models do not assume a predefined model, but instead attempt to derive both the model structure and the parameters from the data. These models are particularly useful when the independent variables do not individually determine the shape of the regression. Many machine learning algorithms, such as decision trees and neural networks, are non-parametric (where the search for network structure is also included as part of the model fitting). The kNN method has the advantage of being simple and transparent in its working. Complex models such as SVM and neural network models are prone to overfitting, and when the performance increase is not significant, these models should not be chosen over a simpler model such as the kNN model. 

For categorical prediction, the skew in the confusion matrices, whereby most people are predicted to be normal weight or overweight, suggests that either (a) there were not enough cases in the other categories (obese I, II or III) in the final dataset, such that it was difficult to build a modest model to predict these groups well, or (b) dietary and physical activity reports largely have people believe that they are eating similar to “normal weight” or “overweight” categories. A possible explanation for the latter (b) is that people in higher BMI classes simply underreport, either because they believe that to be true, or because they are affected by social bias, where they say what they think others want to hear. A categorical prediction, especially for at-risk groups (i.e., post-menopausal women that are overweight or obese), may not be effective unless other algorithms or protocols are developed that can handle the noise in self-reported data. The numerical prediction of body weight using macronutrients may be the approach to use based on these results, as well as the first step towards predicting body weight change using dietary macronutrients. Horner et al. [44] reported that errors associated with the WHI OS dietary data were not associated with BMI, which is in contrast to what we have found here. However, they do report an overall 20.8% instance of underreporting, which is consistent with our findings as well.

Clustering improved the prediction of body weight using self-reported data. The data available for women in clusters that were in some overweight or obese categories were not adequate to build regression models describing their macronutrient intakes. A majority of the clusters indicated a positive association between dietary sugar or carbohydrates and body weight, which is expected. One overweight BMI cluster indicated a positive association between dietary fat and body weight, and another between protein and body weight. A simple conclusion can be that women in these clusters are consuming more of these macronutrients and are therefore, linked to their body weight status. Alternately, these are suggestive of macronutrient “vulnerabilities” especially in the overweight clusters. However, such a speculation would require genetic and other metabolic measurements, such as substrate oxidation following individual macronutrient consumption as support. Clusters that were unable to be represented by regression models suggest a lack of sufficient data to match the variance exhibited, or, similar to the BMI categorical predictions, severe underreporting in these clusters/body weight categories, rendering the models unfit. Another perspective to consider is that ML algorithms are designed to minimize prediction error. However, they can be modified to minimize predictor (independent variable) dimensionality, to better predict classes of outcomes, including underweight and obese, presenting the opportunity for future projects. We also had some success in predicting the cluster each new case would fall into using a classifier, but not well enough to be a reliable predictor. This suggests that either the model needs more independent variables or that the quality of data is poor or noisy, and both of these are likely true. 

A benefit of using epidemiological data for the proposed objectives is the large sample size, which is directly in contrast with earlier prediction models, such as the Harris–Benedict model that used 136 adult males and 103 adult females [7]. However, a primary challenge/limitation we face while answering this question pertains to data obtained using self-reports, such as the food frequency questionnaire, as well as self-reported physical activity, which are memory based and highly prone to bias [45]. Mobile health apps, epidemiological studies and large health-tech companies do use such data, even though their accuracy is controversial [46,47].

Poor quality data can hinder model building, even while using sophisticated tools and approaches to model outcomes. The model is only as good as the data. Specifically, energy intake from food frequency and self-reported data are notorious for producing implausible reports, and several approaches to potentially address this have been presented [17]. The approach presented here incorporates these efforts, to ensure the best effort in achieving the objectives stated. This is an exercise in understanding if the increased power due to a larger sample size, along with sophisticated modeling tools and approaches, can overcome shortcomings in data. It may be that controlled experiments such as the Harris–Benedict are the only way to achieve true energy balance predictions. However, at the population level, the approaches presented in this manuscript may provide us different, otherwise crucial insights as well.

In conclusion, predicting body weight using self-reported dietary, physical activity, demographic, and disease state data is a means to identify “phenotypes” that have different associations between these independent and outcome variables. However, the error associated with such predictions and their inferences have to be carefully considered before using them in applications. An application of this developed approach is use in ongoing epidemiological trials to detect underreporting in self-reported dietary data. Another application is to determine which dietary macronutrient or physical activity component is likely to affect postmenopausal women the most, in a personalized manner, providing an early warning system to watch their dietary intake or physical activity behavior. A recent review paper [48] suggested a framework for personalizing nutrition approaches. Our report is a first step in (a) evaluating a framework for personalizing nutrition using population data and (b) evaluating relationships between dietary caloric variables (macronutrients) and body weight, suggestive of different phenotypes likely responding differently to dietary carbohydrates, fats, or proteins. This suggests that such frameworks/pipelines may be useful to personalize ideal dietary intake levels, beginning at the population level. Multi-center controlled feeding trials, providing an appropriate diet to the appropriate clusters will need to be done in order to evaluate the predictive ability of these models. Further, an ideal future follow-up study would be to use these tools to predict change in body weight using these variables, following a similar approach as presented here. The promise of using machine learning tools to achieve nutritional phenotyping needs to be explored further to set up standard paradigms based on the type of data, origin of data and the researchers’ hypothesis, among several other factors. 

## Figures and Tables

**Table 1:** Feature selection based final list of variables chosen for building models.

Independent Variables	Variable Type
Energy (kcals)	Numeric
Carbohydrates (g)	Numeric
Sugars (g)	Numeric
Protein (g)	Numeric
Fat (g)	Numeric
Fiber (g)	Numeric
Alcohol (g)	Numeric
Age (years)	Numeric
Ethnicity	Categorical
Socioeconomic Score	Numeric
Marital status	Categorical
Vigorous intensity physical activity (h/week)	Numeric
Moderate intensity physical activity (h/week)	Numeric
Mild intensity physical activity (h/week)	Numeric
Height (cm)	Numeric
Overactive thyroid	Binary
Underactive thyroid	Binary
Heart failure	Binary
Angina	Binary
Atrial fibrillation	Binary
Kidney or bladder stones	Binary
Dialysis for kidney failure	Binary
Stomach or duodenal ulcer	Binary
Diverticulitis	Binary
Pancreatitis	Binary
Liver disease	Binary
Multiple sclerosis	Binary
Parkinson’s disease	Binary
ALS Lou Gehrigs disease	Binary
**Dependent Variables**	
Body weight (kg)	Numerical
Body mass index (BMI) (kg/m^2^)	Categorical

**Table 2:** Numerical and categorical approach summaries.

Numerical Algorithms Used	Mean Approximate Error (MAE)	*R* ^2^	Root Mean Square Error (RMSE)
Stepwise linear regression *	10.1	0.22	13.4
kNN	6.98	0.26	8.71
Gaussian SVM regression	6.70	0.30	8.50
Regression tree **	9.53	−0.42	12.1
Neural network	6.90	0.27	8.62
**Categorical Algorithms Using Bagged Tree**	Bagged tree (random forest bag with decision tree learners)	Bagged tree (random forest bag with decision tree learners)	
Ensemble method	Bag	AdaBoost	
Learner type	Decision tree	Decision tree	
Number of learners	30	50	
Maximum number of splits	none	none	
Principle component analysis (PCA)	20/25	20/25	
Accuracy	52.8% (chance >1 out of 6–16.7%)	48.2% (chance >1 out of 6–16.7%)	
**Categorical Decision Tree**			
Maximum number of splits	None		
Split criterion	Maximum Gini Reduction		
Surrogate decision splits	Off		
PCA	20/25		
Accuracy	44.2% (chance >1 out of 6–16.7%)		
**Categorical SVM**			
Kernel function	Radial Basis Function Kernel		
Box constraint level	6		
Multi-class method	1 vs. rest		
PCA	20/25		
Accuracy	54.5% (chance >1 out of 6–16.7%)		
**Categorical kNN**			
Number of neighbors	20		
Distance metric	City block		
Distance weight	Squared inverse		
PCA	20/25		
Accuracy	51.9% (chance >1 out of 6–16.7%)		

* minimum Akaike information criterion (AIC), mixed approach; ** If the correlation between the observed and predicted values is negative, then *R*^2^ is likely to be a negative number, which in this case is true.

**Table 3:** Further exploration of the best-fit kNN model.

Variable	KNN			SVM		
	MAE	RMSE	*R* ^2^	MAE	RMSE	*R* ^2^
With all independent variables	6.98	0.26	8.71	6.70	0.30	8.50
*Dietary variables*						
Energy (kcal)	7.04	8.77	0.26	6.74	8.54	0.30
Protein (g)	7.09	8.82	0.25	6.79	8.60	0.29
Fat (g)	7.06	8.79	0.25	6.75	8.54	0.30
Alcohol (g)	7.01	8.75	0.26	6.77	8.58	0.29
Carbohydrates (g)	7.01	8.73	0.26	6.75	8.54	0.30
Sugar (g)	7.00	8.73	0.26	6.73	8.53	0.30
Dietary fiber (g)	6.99	8.72	0.27	6.74	8.53	0.30
*Physical activity*						
Mild intensity (min)	6.98	8.71	0.27	6.73	8.52	0.30
Moderate intensity (min)	6.99	8.71	0.27	6.73	8.52	0.30
Vigorous intensity (min)	7.00	8.73	0.26	6.73	8.52	0.30
*Blood Pressure (mmHg)*						
Systolic	7.03	8.76	0.26	6.76	8.56	0.29
Diastolic	7.01	8.73	0.26	6.72	8.51	0.30
*Demographics*						
Age (y)	6.99	8.72	0.27	6.74	8.54	0.30
Height (cm)	7.20	8.94	0.23	6.98	8.78	0.26
Ethnicity	7.03	8.76	0.26	6.74	8.54	0.30
Marital Status	6.98	8.71	0.27	6.72	8.51	0.30
Socio Economic Score	7.00	8.73	0.26	6.73	8.52	0.30

**Table 4:** Clusters and their performance, relative to the fit of the complete data model and classifier outputs.

Cluster Number	Average Body Weight (kg) Mean ± SD			Cluster Size (n)	*p*-Value for Correlation between Predicted vs. Actual
All clusters	68.7	±	10.1	48,508	0.07
Cluster number 1	54.4	±	1.4	3613	0.20
Cluster number 2	75.8	±	1.4	4761	0.09
Cluster number 3	67.5	±	1.6	5199	0.42
Cluster number 4 *	80.6	±	1.5	2573	<0.01
Cluster number 5 *	62.8	±	1.1	3611	<0.01
Cluster number 6 *	71.5	±	1.3	4219	0.05
Cluster number 7 *	91.6	±	1.9	3518	<0.01
Cluster number 8 *	66.7	±	1.3	2884	0.02
Cluster number 9	58.8	±	1.2	2112	0.54
Cluster number 10	85.7	±	1.6	1465	0.07

* clusters with significant correlations between predicted and actual body weight.

## Supplementary Materials

Supplementary File 1
